# A Joint Multi-Path and Multi-Channel Protocol for Traffic Routing in Smart Grid Neighborhood Area Networks

**DOI:** 10.3390/s18114052

**Published:** 2018-11-20

**Authors:** Juan Pablo Astudillo León, Luis J. de la Cruz Llopis

**Affiliations:** Network Engineering Department, Universitat Politècnica de Catalunya, 08034 Barcelona, Spain; luis.delacruz@upc.edu

**Keywords:** smart grid, neighborhood area networks, mesh networks, traffic routing, link layer protocols, quality of service

## Abstract

In order to improve the management mechanisms of the electric energy transport infrastructures, the smart grid networks have associated data networks that are responsible for transporting the necessary information between the different elements of the electricity network and the control center. Besides, they make possible a more efficient use of this type of energy. Part of these data networks is comprised of the Neighborhood Area Networks (NANs), which are responsible for interconnecting the different smart meters and other possible devices present at the consumers’ premises with the control center. Among the proposed network technologies for NANs, wireless technologies are becoming more relevant due to their flexibility and increasing available bandwidth. In this paper, some general modifications are proposed for the routing protocol of the wireless multi-hop mesh networks standardized by the IEEE. In particular, the possibility of using multiple paths and transmission channels at the same time, depending on the quality of service needs of the different network traffic, is added. The proposed modifications have been implemented in the ns-3 simulator and evaluated in situations of high traffic load. Simulation results show improvements in the network performance in terms of packet delivery ratio, throughput and network transit time.

## 1. Introduction

Electric energy is currently an essential resource all around the world. With the increase in the use of new technologies in all sectors of human activity, it is easy to predict that the consumption of this type of energy will grow considerably in the near future. For this reason, great research and development efforts have been made, with the aim of improving the generation processes, transport networks and storage systems for this energy. The smart grid networks are the result of the work carried out to obtain improvements in the management, operation and maintenance of the transport infrastructure, as well as in the efficiency with which the energy is used. Their main objective could then be considered as achieving the best use of electrical energy through an improvement in the management and maintenance of the energy sources and the transport infrastructure. At the same time, new services are offered to both supplying companies and consumers.

With these objectives in mind, one of the main advances is being made in the improvement of the data networks associated with the electricity transport infrastructures. These data networks are responsible for carrying and delivering all the control, management, maintenance, and security information of the electricity network infrastructure, as well as the applications that allow a better management of the available resources. The data networks are therefore a fundamental part of the smart grid, and so, their reliability, availability, and security have to be guaranteed in all situations, taking into account that they can stop providing correctly their service due to intrinsic (hardware, software, communications protocols, etc.) or extrinsic (weather conditions, malicious agents, terrorist attacks, etc.) failures [1].

The smart grid data communication network is made up of several subnetworks. The different Smart Meters (SM), devices, and other utilities present inside the customers’ homes are interconnected by the Home Area Network (HAN). These HANs are in turn interconnected through the Neighborhood Area Network (NAN), and finally, the information can reach the control centers through a Wide Area Network (WAN). To implement all these subnetworks, different technologies can be chosen. Selectable technology standards for HANs can be, among others, IEEE 802.15.4 (Low-Rate Wireless Personal Area Networks (LR-WPAN)) or IEEE 802.11 (Wireless Local Area Networks (WLAN)). For NANs, Power Line Communication (PLC) technologies or standards such as IEEE 802.15.4g or IEEE 802.11s (Wireless Mesh Networks) can be considered.

Guaranteeing that the quality of service offered is that required by every application, while maintaining a high efficiency in the use of resources, is one of the most important issues to be taken into account both during the planning process and during the maintenance and operation of the network. It is therefore essential to have deep knowledge of the services that will be provided. These issues are especially important when working with smart grids, given the critical importance of the power grid infrastructure. The offered services belong to very different types, and therefore, their service quality requirements are also different [2]. Generally speaking, most smart grid applications have strong security and reliability requirements. However, their bandwidth needs, as well as their behavior in high packet losses or high latency situations can be very different. Thus, some applications, such as Substation Automation Systems (SAS) or overhead transmission line monitoring, present very strict requirements in terms of latency, but they are not as demanding in terms of bandwidth. On the other hand, demand response management or Advanced Metering Infrastructure (AMI) generally consumes more bandwidth, but can allow greater delays.

In the context of WLANs standardized by the IEEE, the proposal for multi-hop mesh networks was published in 2011 as Amendment Number 10 to the 2007 general standard, with the name of IEEE 802.11s [3]. In the revision of the general standard published in 2012, as well as in the current revision [4], mesh networks have been directly incorporated, although a large number of researchers continue to refer to them as IEEE 802.11s mesh networks. The main differentiating characteristic of this type of networks is that, from the upper layer point of view, all the stations appear as connected at the MAC level, although they might not be within the range of coverage. To make this possible, a layer 2 path search mechanism called the Hybrid Wireless Mesh Protocol (HWMP) was designed.

In this article, some modifications on the basic mechanisms and protocols used by the IEEE 802.11 mesh networks are proposed, in order to improve their performance when using this technology as the implementation of the smart grid NANs. Mainly, a new multi-path mechanism is proposed and implemented in conjunction with a multi-channel allocation of the different available paths. These paths are assigned differently according to the quality of service demanded by every traffic. With this strategy, we intend to take better advantage of the available network resources, guaranteeing an adequate service to as much traffic as possible. In this way, the supplier companies can obtain greater benefits by being able to provide service to a higher number of clients or applications, while at the same time, they can offer more competitive prices to their customers. The study and comparison of the benefits obtained is based on simulations carried out with the ns-3 network simulator [5]. Thanks to the improvements obtained, especially in situations of high load, NAN networks could offer service to a greater volume of traffic and also allow a correct and differentiated quality of service for each application. In this way, new development challenges are created for both device manufacturers and electricity supplier companies, which will be able to offer new and better services to their customers. From our point of view, these new services should have a major impact on greater efficiency in the use of electricity and a faster and improved reaction in front of emergency situations.

Smart grid networks have attracted the attention of numerous researchers in recent years. Among these investigations, several proposals have been presented in order to improve the benefits offered by the NANs, where both wired and wireless technologies have been taken into account [6]. Within the wired technologies, PLC stands out especially in this environment due to its ability to use the existing infrastructures. However, the available bandwidth with this technology is quite limited, and it also presents drawbacks when data signals must pass through electrical transformers. When the number of nodes in the network grows, as well as the bandwidth needs, other technologies must be considered. In this sense, wireless networks in general [7,8] and wireless multi-hop networks in particular [9,10] present a series of advantages that make them ideal candidates. For instance, they do not require previous infrastructures, and their bandwidths are constantly increasing. Besides, they have a great flexibility to modify the network topology and to take advantage of multi-channel and multi-path mechanisms that increase their performance in terms of, among others, availability, packet delivery ratio or network transit time. For these multi-hop wireless networks, a new and precise analytical model, which takes into account the hidden nodes problem, has been presented in [11].

In [12], an enhancement of the Optimized Link State Protocol (OLSR) in order to satisfy the required level of reliability in NANs is presented. The possibility of offering an adapted quality of service to the different data traffics transmitted through the network is taken into account. To this end, the authors proposed the use of a combination of different basic metrics: Expected Transmission Count (ETX), Minimum Delay (MD) and Minimum Loss (ML). They chose Relevant Link Metric Types (RLMTs) for each application (traffic type), assigned different weights to each of them, and used a pruning technique to reduce the number of considered paths to a given destination. The best link to send each traffic was then calculated by means of an AHP (Analytical Hierarchy Process) algorithm. The proposal was evaluated by means of ns-2 simulations, over a usual network environment consisting of a grid of smart meters transmitting (receiving) information to (from) a data concentrator and taking into account four basic CBR traffic types. Moreover, the topology was modified by increasing the number of smart meters (from 25 to 64) and changing the data concentrator position. The network performance was measured in terms of dropped packets, packet delivery ratio and average delay, showing a better behavior when compared to a basic OLSR implementation. The same authors previously presented in [13] a performance evaluation and comparison of the OLSR and HWMP (IEEE 802.11s) routing protocols, together with a classification of the main AMI application traffic.

A multi-gate communication network, based on IEEE 802.11s, was proposed in [7] for smart grids. The authors took into account the possibility of having more than one node acting as a gateway, together with a real-time traffic scheduling and a multi-channel-aided routing protocol. Besides, the authors proposed a heuristic backpressure scheme, where every node evaluated the state of its neighbors before selecting one of them as the best next hop, which implies that some information (the backpressure metric) must be periodically exchanged between nodes. Otherwise, to avoid loop problems, a hop-count limit was imposed on the data packets. Besides, in order to reduce the effect of co-channel interference, a multi-channel protocol was also introduced. To evaluate the proposals, three simulation scenarios were taken into account: (a) three separated sub-networks where every one had its own gateway, (b) a multi-gateway network where the three previous networks shared their three gateways and where the nodes were uniformly distributed, and (c) the previous configuration, but with an asymmetrical distribution of the nodes. The results showed the better behavior offered by the proposed backpressure scheme in terms of overall throughput, average end-to-end delay, and adaptation to malfunctioning nodes. On the other hand, the benefits of the multi-channel protocol were also clearly shown.

A cross-layer mechanism that combines information from the physical, MAC, and network layers was presented in [14]. Based on that mechanism, the authors defined a new routing metric (Expected Path Throughput (EPT)) and a distributed routing protocol, which was evaluated with the help of the ns-2 simulator. The results showed the good behavior of the proposal when compared with other classical metrics and protocols.

In [15], the authors proposed the HWMP-NQ protocol, a modification of HWMP to ensure the Needed Quality of Service (QoS) of several smart grid traffic types. To this end, the airtime link metric was modified by considering the packet size and the transmission rate. However, the needed number of channel measurement could be excessively increased. To avoid this, a frame error rate computing algorithm based on a single measurement was also proposed. Besides, the benefits provided by a multi-gateway backup routing scheme were also analyzed. Moreover, to reduce the routing overhead in case of link failures, a modification of the path error mechanism was introduced. To evaluate the benefits of their proposals, the authors built classical NAN grid topologies with the help of the ns-3 simulator and ran multiple simulations to measure the average throughput, packet delivery ratio, end-to-end delay, and routing control information overhead. The results showed the benefits of the multi-gate routing scheme presented in [7] and the HWMP-NQ protocol, in front of the classical HWMP implementation, for different NAN grid sizes (from 9–64 smart meters). What is more, the influence of the node failure rate was also studied, showing that the performance improvements obtained with the authors’ proposals increased when that failure rate was higher.

In order to improve the network throughput and reliability, another modification of the airtime link metric calculation method was presented in [9]. One of the contributions of this work was to give more importance to the upstream transmission status (from smart meters to the concentrator), since most data were transmitted in this direction. Besides, a modification of the path selection mechanism was provided to avoid the classical problem of route fluctuation. With this modification, not only the current airtime link metric value, but also its variations were taken into account to select (or not) a new route between two network nodes. ns-3 simulations were presented to show the achieved benefits in terms of packet delivery ratio, end-to-end delay and data retransmission count. The results also highlighted the need for congestion control mechanisms when the network size was increased.

Some of the same authors of [9] performed in [10] a study of the HWMP routing protocol, with the goal of identifying its weakness, both from the HWMP protocol itself (route instability and route recovery) and from the integration with smart grid networks (oversimplified calculation of airtime link metric and the need for traffic differentiation). Here, a modification of the airtime link metric computation was also proposed, as well as a proposal for the path selection mechanism. Besides, to get a better performance in terms of packet losses, reserve routes were stored in the network nodes. This idea gave rise also to a reduction in the traffic management traffic needed when a path was broken. Moreover, in order to provide a better quality of service to some applications, a delay-tolerant traffic management method based on the concept of delay-tolerant networking was proposed. The improvements obtained with the application of these new solutions to the protocol, called HWMP-reliability enhancement (HWMP-RE), were checked and shown by means of ns-3 simulations. Grid topologies were considered, from 9—49 nodes, where every node generated traffic (belonging to seven different applications) to two root mesh stations (gateways). HWMP-RE was compared with the basic HWMP and with the previous proposal in [9], showing a better behavior in terms of packet delivery ratio, end-to-end delay, number of PERR/PREQ generations, throughput and reliability.

Other proposals based on the modification of the HWMP metric can be found in [16,17]. In [16], a QoS-aware and load-balance routing scheme was proposed, which was complemented with an EDCA-based adaptive priority adjustment scheme, with the goal of satisfying the QoS requirements of different NAN applications. The modification proposed for the airtime link metric consisted of including the packet size and calculating the frame error rate separately for the different NAN applications. Besides, to avoid congested paths, the queuing delay was also added to the metric. What is more, the dynamic adjustment of the packet priority allowed a better resources utilization under low load conditions and improved the reliability under heavy load conditions. ns-3 simulations were carried out to evaluate the obtained performance, which showed an increase of both the packet delivery ratio and the throughput, as well as a reduction of the average end-to-end delay. The network scenario consisted of a grid topology where the number of nodes varied between nine and 64.

On the other hand, the metric modification proposed in [17] (Interference-Aware Expected Transmission Time (IAETT)) was oriented to reduce the impact caused by inter- and intra-flow electromagnetic interferences. Besides, traffic differentiation was also considered. Based on this metric, an interference-aware QoS routing protocol was proposed and evaluated. The performance evaluation was carried out again by ns-3 simulations, over a scenario consisting of 100 nodes arranged in a 10 × 10 regular grid, where both the gateways (nine nodes) and the traffic generating nodes were randomly chosen. Results showed the improvements obtained in terms of average end-to-end delay and packet delivery ratio.

As already mentioned, this paper presents a new proposal for improving performance in smart grid NANs when using IEEE 802.11 mesh network technology. Although the modification of the routing metrics is a good idea to differentiate the service offered to different traffic in the network, we preferred to maintain the basic airtime link metric and focused our efforts on the modification of the mechanism used by the HWMP protocol for the selection of the most appropriate path each time a data packet must be (re)transmitted. By its own nature, the default metric informs about the congestion state of the different network areas, which is the most relevant measure for our approach. Moreover, it is important to keep in mind that working with more complicated metrics usually leads to higher CPU and memory requirements in the network nodes, as well as to protocols that generate more network control traffic. In this way, a modification of the HMWP protocol is proposed and implemented, to allow an efficient selection of paths among multiple possibilities, depending on the service quality needs of the different traffic flows. The proposed mechanism is complemented with the assignment of different frequency channels to each available path. In addition, to avoid packet losses due to the formation of unwanted loops, the proposed technique is combined with a criterion of the minimum number of hops when choosing the paths. This technique reduces the number of selectable paths, but avoids the need to use packet hop counters (which are used by the nodes to discard packets after a given number of hops, with the added disadvantage of using network resources for a certain number of retransmissions in a completely useless way). On the other hand, as will be seen in the Results Section, we considered it of great importance to provide not only the average values of the performance parameters under study, since this way, the real network performance is not obtained and would probably lead us to an erroneous network planning.

The rest of the paper is organized as follows. The next section presents the modifications proposed for the HWMP protocol. Details about the multi-path and multi-channel mechanisms implementation are provided, as well as the route selection and assignment algorithms. Section 3 presents and analyzes the results obtained through the simulations, and finally, the conclusions, as well as the future lines of our research are summarized in Section 4.

## 2. A Generic Multi-Path and Multi-Channel Proposal for HWMP

**Figure 1:**
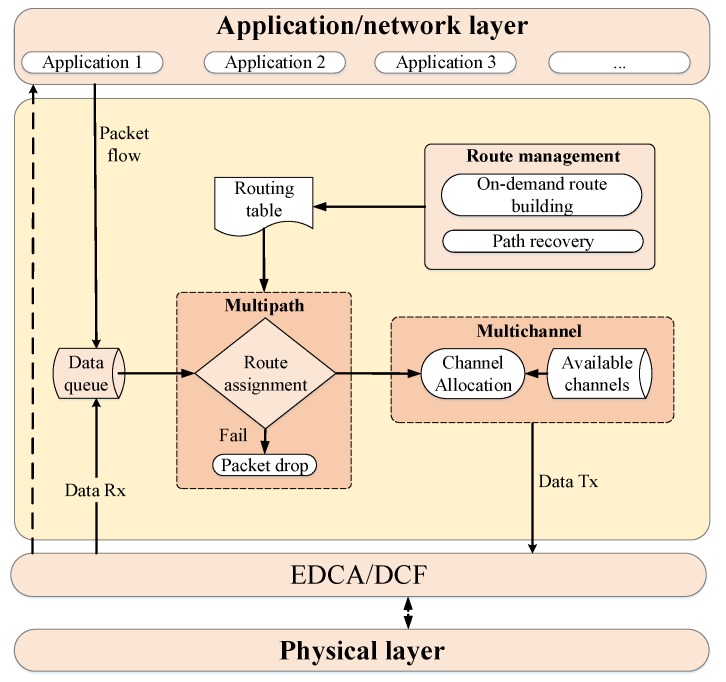
General view of the multi-path and multi-channel modules inclusion in HWMP.

Figure 1 shows the proposed modified structure for the HWMP algorithm. On the one hand, the MSTAs are capable of storing multiple paths to every destination node in their routing tables. These paths are classified by a path selection policy with the objective of sending the data traffic with the highest priority over the best paths. On the other hand, to reduce the level of interference between MSTAs and increase the network performance, a different channel is assigned to each available path. Besides, a different channel will be reserved for control packets. In order to add these multi-path and multi-channel functionalities to the default HWMP protocol, several mechanisms are proposed. To evaluate their performance, all the proposals have been programmed and included in the basic ns-3 IEEE 802.11s module [5].

### 2.1. Multipath Proposal and Implementation

HWMP establishes by default a single path between the source and destination nodes. The purpose of this section is to modify the protocol to obtain and take into account all the possible paths between two nodes. As already said, these available paths will be assigned to the different applications (traffic types) depending on their priority. Therefore, the modules related to the route management, routing table and route assignment have been modified.

#### 2.1.1. Route Management

**Figure 2:**
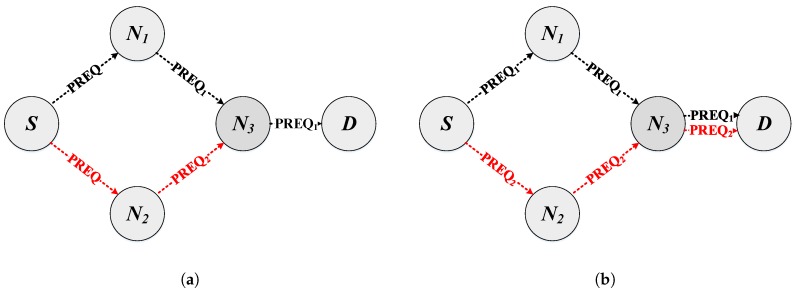
Modification to the acceptance criteria when the intermediary nodes receive a PREQ message. (**a**) HWMP. $N_{3}$ receives, processes and forwards the $PREQ_{2}$ message if and only if it has a better metric than $PREQ_{1}$. (**b**) Multi-Path Multi-Channel (MPC)-HWMP. $N_{3}$ receives, processes and forwards $PREQ_{2}$ to maintain multiple paths.

To allow the existence of more than one path between each pair of nodes, it is first necessary to modify the acceptance criteria for both PREQ and PREP packets. Table 1 summarizes all the functions and variables needed for the new PREQ-PREP mechanism. As shown in Algorithm 1, first of all, the most relevant fields are extracted. Then, the metric value for every path is updated. Next, the algorithm has to validate the message, that is verify if the current message has more recent information (the sequence number of the current message is greater than the previous one, $SN_{C_{OA}}>SN_{P_{OA}}$) or if there is a better metric when the sequence number is the same ($SN_{C_{OA}}=SN_{P_{OA}}$). By default, HWMP updates the route to the originator address and replaces the previous route when the sequence numbers of multiple received PREQ messages are equal, but one of them has a better metric. For instance, in the example shown in Figure 2a, the source node S generates a PREQ message to find a path to the destination node D. Two instances of this PREQ (first $PREQ_{1}$ and then $PREQ_{2}$) are received by $N_{3}$ from two different nodes (paths), and only the one with the best metric will be retransmitted to D ($PREQ_{1}$ in the figure). In our proposal, the $PREQ_{2}$ message is also retransmitted (Figure 2b), because we need to compare not only the sequence numbers and the metrics in order to validate a PREQ message, but also take into account the previous (retransmitter) node in the path ($f_{r}$ in Table 1 and Algorithm 1). The objective is to maintain multiple paths to the originator address (OA) through different $f_{r}$ nodes. After validating the message, the routing table is updated (specifically, the table entries related to the neighbor and to the source nodes). In addition, if there were queued packets for the new or updated route, they would be immediately transmitted. In the case that the PREQ destination address is the one of the receiving node, it means that a route has been found. Therefore, this node transmits directly a unicast PREP message towards the S node.

**Figure 3:**
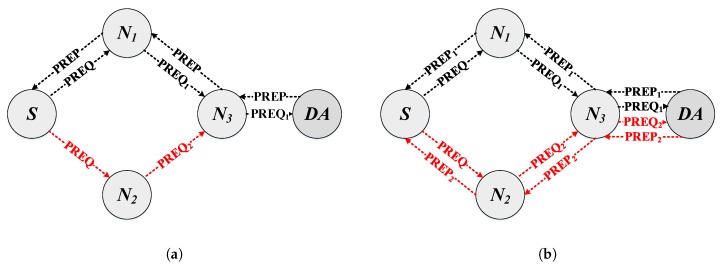
Modification to the acceptance criteria when the destination node receives a PREQ message. (**a**) HWMP. The destination node replies with a unicast PREP message to the source node. (**b**) MPC-HWMP. The destination node replies to all received PREQ with unicast PREP messages to the source node through its different paths.

In the same way, with the default algorithm, the destination node responds with a PREP message to the source node if and only if the PREQ received has a better metric than the previous ones (Figure 3a). However, in our implementation, a PREP message is sent, although the received PREQ has a worse metric. This action allows propagating not only the best path to the source, but also having several paths with different metric values (Figure 3b).

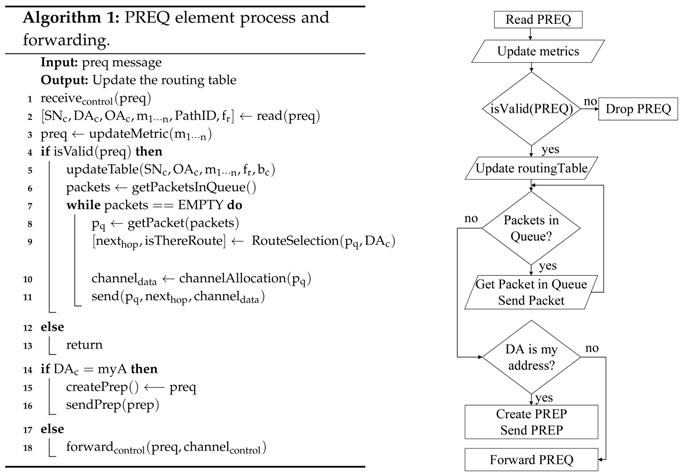



The main modifications for the reception, processing and forwarding of a PREP message are similar to those explained for the PREQ messages and are detailed in Algorithm 2. On the other hand, the criteria for the retransmission of the PREP messages towards the node that originated the PREQ message must consider the multiple paths created and not take erroneous paths. In other words, each PREP message must know which was the path that its corresponding PREQ message took. To this end, a field has been added both to the PREQ-PREP messages and to the routing table (PathID), which allows the nodes to obtain the correct path (destination address) for each PREP message that must be generated or forwarded. For instance, in the example shown in Figure 3, $N_{3}$ makes use of this field to route $PREQ_{1}$ and $PREQ_{2}$ to their corresponding nodes correctly. 
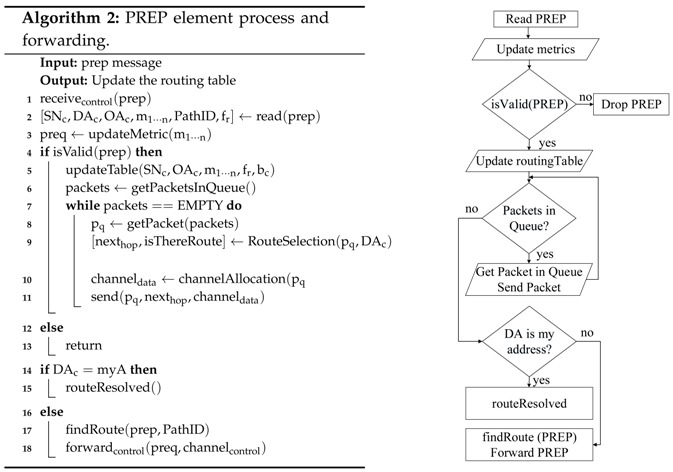


### 2.2. Multi-Channel Mechanism

The objective of implementing multi-channel techniques is to increase the network performance mainly in demanding situations, that is when a high amount of data traffic is being transmitted by the network. Our proposal implements traffic differentiation in the following way. First, we have used different channels for control and data traffic, where just one channel is assigned to the control traffic (route management). On the other hand, as explained in the previous section, the different data traffic is mapped to the available paths depending on its priority, and a different channel is assigned to every path. Thus, each channel will have a specific metric value, which will be useful for future routing decisions.

**Figure 4:**
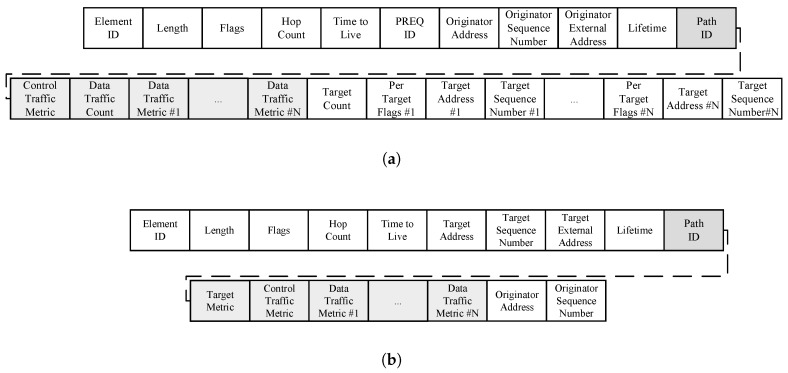
Path request and path reply modifications. (**a**) Path request. (**b**) Path reply.

The airtime link metric is cumulative and updated in each node by the Path Request (PREQ) and Path Reply (PREP) messages. Therefore, to propagate the ALM metrics according to their respective channel, it is necessary to make modifications to the structure of the PREQ and PREP messages, as can be seen in Figure 4. The modification consists of the inclusion of the path identifier field, the metric value for the control channel, the number of available paths (channels), and the metric value for each of them.

By default, the HWMP protocol performs a broadcast of PREQ messages for path discovery through all the available interfaces in the node. Therefore, with the multi-channel implementation, the number of broadcast messages could be increased excessively. This is the reason why a specific channel has been defined for control messages, thus avoiding a high and unnecessary load on the data channels.

To implement the path/channel allocation, the applications are marked from the source with an Enhanced Distributed Channel Access (EDCA) category [4]. EDCA distinguishes between four types of traffic according to their QoS needs. The types of traffic with higher priority are mapped to the highest categories of EDCA (voice or video), and vice versa. Therefore, intermediate nodes are able to select the next hop node from their routing table among the multiple available paths to the destination and transmit the application traffic over the correct channel.

### 2.3. Routing Selection and Assignment

The proposed mechanisms modify the default HWMP routing table. On the one hand, the number of entries in the table will be higher, due to the availability of multiple paths for each destination address. In addition, the number of fields of each entry will also be higher to allow the appropriate path selections. The added fields are summarized in Table 2.

The general route assignment tasks performed by the network nodes every time they have to (re)transmit a packet are detailed in Algorithm 3. First, the node extracts the following parameters from the packet header: source node, destination node, access category, and time to live. Then, the algorithm verifies if there is an available route. In the affirmative case, the next hop node is obtained by means of the route selection algorithm (Algorithm 4), considering the destination node and the access category, and finally, the transmission is assigned to a specific channel. In the case that there is no route, the packet is queued, and the path discovery mechanism is activated. This mechanism tries to obtain the route a fixed number of times, and if that threshold is exceeded, the route to that destination is considered invalid and the packet is eliminated.

**Figure 5:**
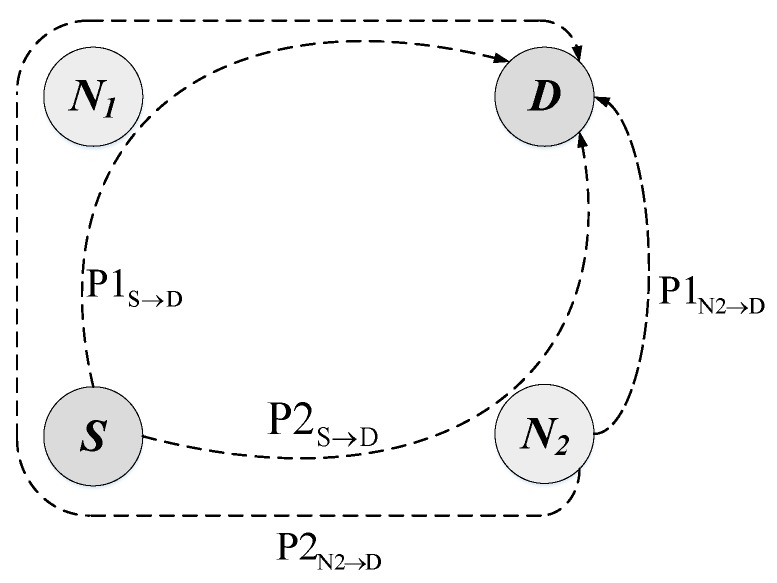
Possible loop creation for low priority packets.

As can be seen in Algorithm 4, the route selection process first searches all available paths according to the destination address and then deletes the routes that have expired. Later, these paths are sorted from the best to the worst according to the metric, and then, they are resorted taking into account the number of hops to the destination. Our implementation considers the number of hops to avoid the creation of undesired loops, as will be explained with the help of Figure 5. This figure represents a simple scenario with four nodes: source (*S*), destination (*D*) and two intermediate nodes ($N_{1}$ and $N_{2}$). The source node has two available paths to send its packets to the destination, $P1_{S\to D}$ through $N_{1}$ and $P2_{S\to D}$ through $N_{2}$. Suppose that at a given moment, the metric value of $P1_{S\to D}$ is better than the metric value of $P2_{S\to D}$. Therefore, according to the multi-path mechanism, high priority packets will be sent to $N_{1}$ and low priority packets will be sent to $N_{2}$. Similarly, $N_{2}$ has two available paths to *D*, one with a better metric value directly to D, $P1_{N_{2}\to D}$, and another with a worse metric value $P2_{N_{2}\to D}$ through *S*. In this way, low priority packets would be sent back to S, building a loop from which they would never leave. To avoid this problem, the criteria of the minimum number of hops is also taken into account, so that $N_{2}$ will never use the $P2_{N_{2}\to D}$ path, sending all the packets with destination in *D* directly to *D* regardless of their priority. This way, the source node *S* is allowed to use its two available paths, sending high priority packets through $N_{1}$ and low priority packets through $N_{2}$, but $N_{2}$ must use always the same path to *D*. As previously said, this mechanism reduces the number of available paths, but avoids the creation of undesired loops, eliminating the need for packet hop counters and unnecessary retransmissions which consume network resources in a completely useless way. 
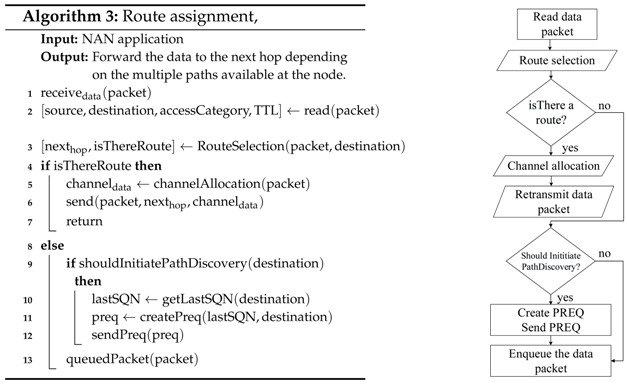
 
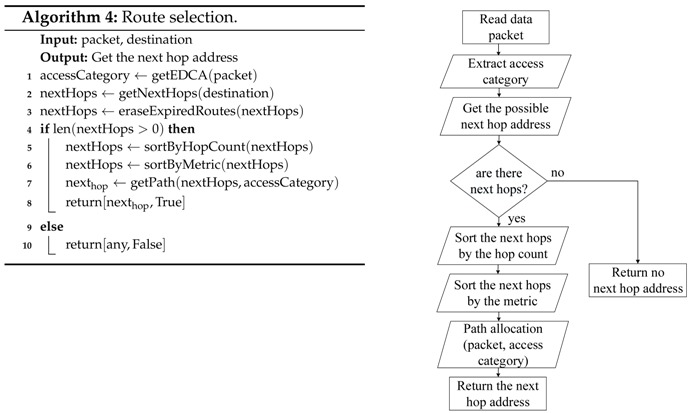


## 3. Results and Discussion

In this section, the evaluation of the proposed modifications for the HWMP protocol is presented. As said before, we chose the ns-3 simulator to carry out the performance evaluation. It includes the default 802.11s model, which was modified to include the proposed mechanisms. In the following sections, the scenario used for our simulations is presented, together with the simulation parameters and the obtained results.

### 3.1. Simulation Environment

**Figure 6:**
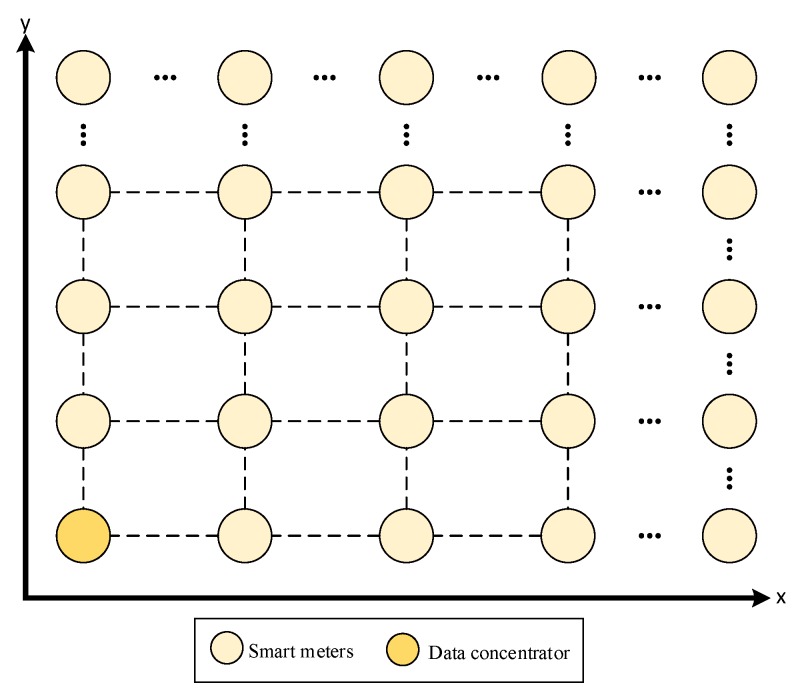
Scenario under consideration.Smart grid architecture.

The scenario for the evaluation of our proposal is shown in Figure 6. This scenario consists of a grid topology where the transmitted data traffic is bidirectional. That is, home users (smart meters and other home devices available at the HANs) transmit different applications (traffic types) to the data concentrator, such as periodic billing data (meter reading), Electric Vehicle (EV) charging information, and home energy, among other applications. On the other hand, the data concentrator is capable of transmitting demand response information to home users, with the aim, for example, of adjusting the energy consumption during peak hours. In the simulations, the number of nodes in the grid is a variable parameter, the data concentrator is located in the bottom left corner, and all applications are running simultaneously in every HAN.

### 3.2. Application Layer

Table 3 indicates the general parameters used in the simulations, where for each grid size (number of NAN nodes), 21 different simulation runs were carried out. Each run was configured with different random seeds in order to obtain confidence intervals for the chosen performance measures: packet delivery ratio, throughput, network transit time, and routing table size. To ensure the network topology shown in Figure 6, we chose 80 meters as the grid distance between nodes. With this value and the propagation model parameter values, each node is only able to establish connections with the neighbors located on its sides.

The applications that will be transmitted over the NAN network are detailed in Table 4, where they are grouped into four types of traffic according to the EDCA categories and the quality of service requirements. The table shows also the distributions and average values selected for the packet size and for the interarrival time, where two main distributions were considered. On the one hand, for periodical traffic types where applications generate packets of constant size at regular time intervals, the distributions were considered as deterministic. On the other hand, for traffic types based on events or by variable rate applications, an exponential distribution was selected, which can be better adjusted to the combination of this type of applications. To implement the latter distribution, the default application module of the ns-3 simulator was also modified. In addition, we considered it interesting to analyze the network performance in two different load conditions. Thus, two different sets of values were selected for the packet generation rate. Firstly, a relatively high Network Load (NL1) was considered and, secondly, a network load that caused an extreme congestion situation (NL2).

### 3.3. MAC and Physical Layers

The IEEE 802.11 standard defines the methods to initiate, maintain, and close the bidirectional links between mesh STAs and also establishes by default the Hybrid Wireless Mesh Protocol and the airtime link metric. Table 5 and Table 6 present the parameters configured in the simulator for the MPM and HWMP protocols, where, among others, the following variables are defined: maximum thresholds to consider invalid links, maximum number of neighbors (peer links) allowed, the reactive mode of HWMP, lifetime of the reactive routing information, and the conditions to indicate a route as unreachable. As mentioned in the previous section, nodes must be allowed to establish links only with the neighbors at their sides. To reinforce this, the maximum number of peer links per node was set to four. On the other hand, the 802.11s model implemented in the ns-3 simulator considers a link as not valid if the consecutive number of lost beacons achieves a configurable threshold (maxBeaconLoss in Table 5). A value of 20 lost beacons was selected for this parameter. In addition, when a station is unable to transmit to its peer a number of successive data frames, the ns-3 implementation by default closes their peer link. This parameter and the other variables presented in Table 5 and Table 6 were configured with their default values. These selections do not affect the performance evaluation carried out, since the values are the same for both compared protocols.

Table 7 presents the configured values for the physical layer, detailing among others the following parameters: 802.11a as the selected physical layer, frequency channels for control and data traffic, and the propagation model. Except for the number of control and data channels and their frequencies (which have been defined in this proposal), well-known values were chosen for the rest of the parameters in Table 7, which were used in most smart grid NAN simulations. As previously said, this selection does not affect the comparison between the protocols.

### 3.4. Results

In this section, we present and evaluate the obtained results. Although the ns-3 simulator provides some tools for data analysis, they are mainly designed to work with protocols that operate at the network layer. As this work is focused on a protocol (HWMP) that operates at the data link layer, a new tool was designed. In the following sub-sections, the obtained results are evaluated in terms of packet delivery ratio, network transit time, throughput, routing tables size, and control channel utilization. All the modifications made to the HWMP ns-3 model can be downloaded from [18].

#### 3.4.1. Packet Delivery Ratio

**Figure 7:**
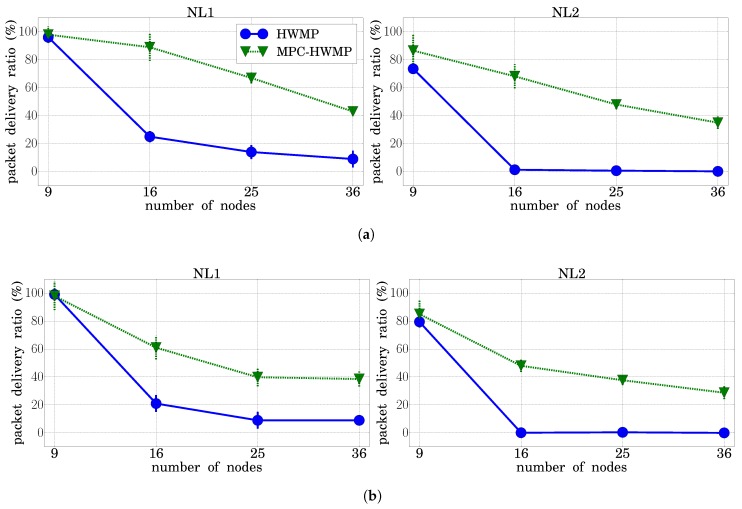

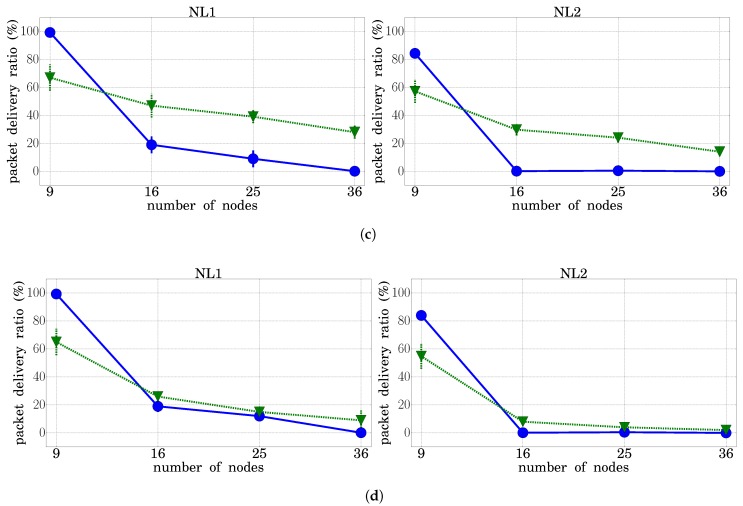
Packet delivery ratio (HWMP vs. MPC-HWMP). (**a**) Traffic Type 1; (**b**) Traffic Type 2; (**c**) Traffic Type 3; (**d**) Traffic Type 4.

We compared the Hybrid Wireless Mesh Protocol (HWMP) algorithm with our proposed extension Multi-Path Multi-Channel Hybrid Wireless Mesh Protocol (MPC-HWMP) in terms of packet delivery ratio (and its 95% confidence interval) for different grid sizes (from 9–36 nodes). The PDR defines the relationship between the number of successfully received packets and the total number of transmitted packets. Figure 7 shows the results for the four traffic types considered (Figure 7a–d, respectively). Besides, for every traffic type, the graph on the left side shows the results under the load conditions NL1, while the one on the right shows them for NL2. The results confirmed that, as the size of the network increased, the PDR decreased for the four traffic types and for the two network load conditions considered. As can be seen, under the NL1 conditions, the network started to be very loaded for a number of nodes greater than 16, while the NL2 conditions led to a total saturation and a PDR value equal to zero when the basic HWMP was used. However, the PDR decrement was much lower with MCP-HWMP. In addition, this figure highlights that, when using MPC-HWMP, the traffic with higher priorities, which uses the best available paths, received a better service from the network than that with lower priorities.

#### 3.4.2. Throughput

**Figure 8:**
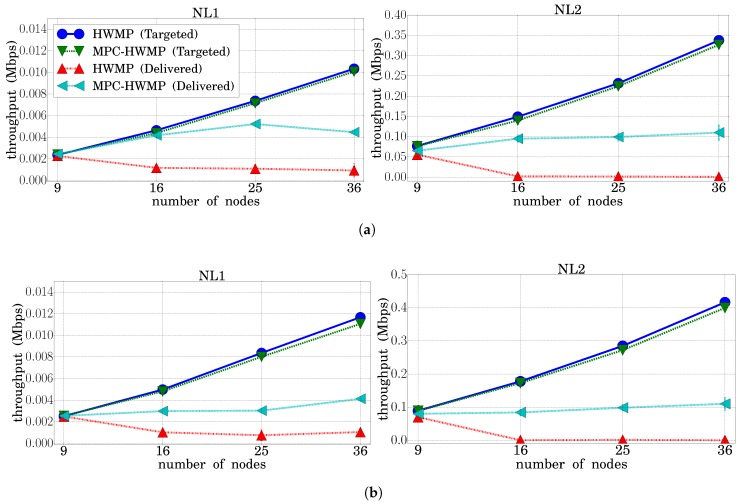

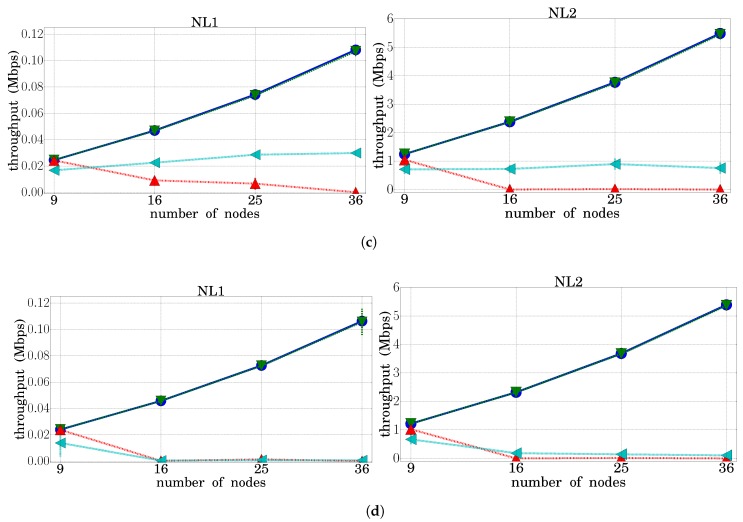
Throughput (HWMP vs. MPC-HWMP). (**a**) Traffic Type 1; (**b**) Traffic Type 2; (**c**) Traffic Type 3; (**d**) Traffic Type 4.

In this section, the results obtained for the network throughput are presented. The throughput represents the number of bits per second transmitted correctly, and it is a performance parameter that complements the PDR offered in the previous section. Figure 8 shows on the one hand the “targeted” throughput, which consists of the bits per second generated by all the applications. As can be seen, this throughput was the same regardless of whether the protocol used was HWMP or MPC-HWMP, and it was higher for the NL2 load conditions. However, the throughput correctly delivered to its corresponding destination was higher when the protocol used was MPC-HWMP. In particular, it can be checked that the throughput delivered with HWMP tended to zero when the network size was equal to or greater than 16 nodes, which is consistent with the results already commented on for the PDR.

#### 3.4.3. Network Transit Time

The network transit time is the time that packets need to go from their source to their destination through the intermediate nodes. For this parameter, instead of offering just the average value, which could hide relevant variations in the service offered to different packets, we have considered it of importance to offer also the percentile value (specifically, the 95th percentile was chosen). On the other hand, to show the existing differences depending on the specific location of each node with respect to the data concentrator, the results are provided separately for the nodes that were in the best and worst situation, that is for the nearest node and for the farthest node to the data concentrator.

**Figure 9:**
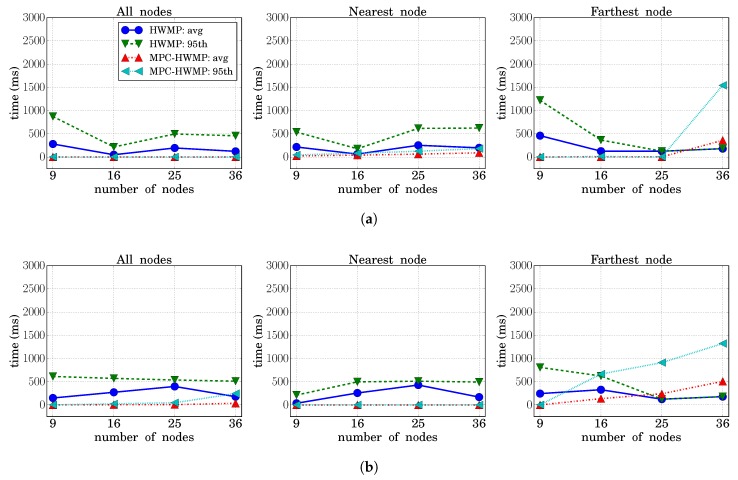

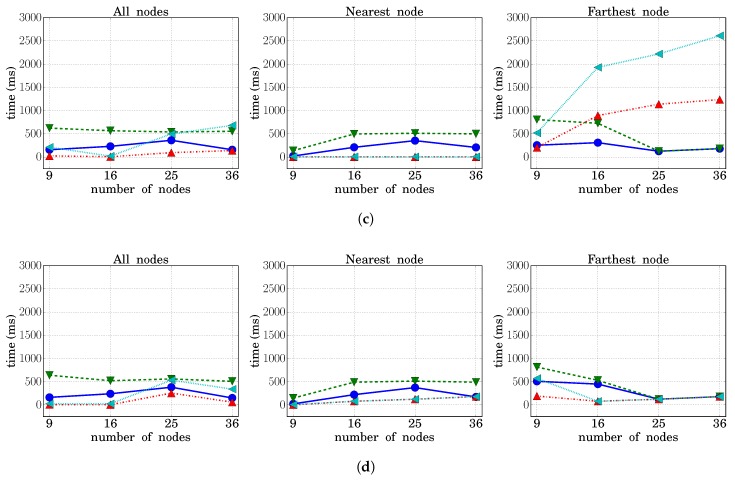
Network transit time (HWMP vs. MPC-HWMP). (**a**) Traffic Type 1; (**b**) Traffic Type 2; (**c**) Traffic Type 3; (**d**) Traffic Type 4.

Figure 9 compares the average and the 95th percentile values for the HWMP and MPC-HWMP cases, when the network is working under load conditions NL2. For each traffic type, the graph on the left side shows the averaged values considering all the network nodes, the graph on the center shows the values considering only the nearest node to the data concentrator, and the graph on the right side takes into account only the values obtained for the farthest node. The general tendency of these time values should be to grow as the network size is increased, and it can be observed that network transit times were smaller when the proposed MPC-HWMP was used. However, paying more attention to the particular cases, some details have to be discussed. First, it can be observed that in an extreme congestion situation and with the basic HWMP protocol, the transit time values shown for the farthest node became smaller instead of greater when the network size was incremented (this also occurred, although to a lesser extent, for other nodes and with NL1 load conditions). This is due to the fact that the vast majority of packets were being lost (remember that the PDR was practically zero), so that only the values of the few packets that arrived correctly were taken into account. These packets have found the network in an instantaneous (and transitory) low load situation, and therefore, its transit time was small. However, looking at the MPC-HWMP protocol, where a significantly greater number of packets were transmitted correctly, the value of the network transit time grew with the number of nodes, as expected. Here, we have an exception again, since for Traffic 4 (lowest priority), the high amount of losses (see Figure 7) caused the same effect as for the HWMP protocol.

On the other hand, looking at the nearest node, the growth in the value of the transit time as the network size was increased was smoother because this node in particular was affected much less by the increase of the network size. For all cases, it can be observed that the best performance in terms of transit time was obtained with MPC-HWMP.

#### 3.4.4. Routing Table Size

**Figure 10:**
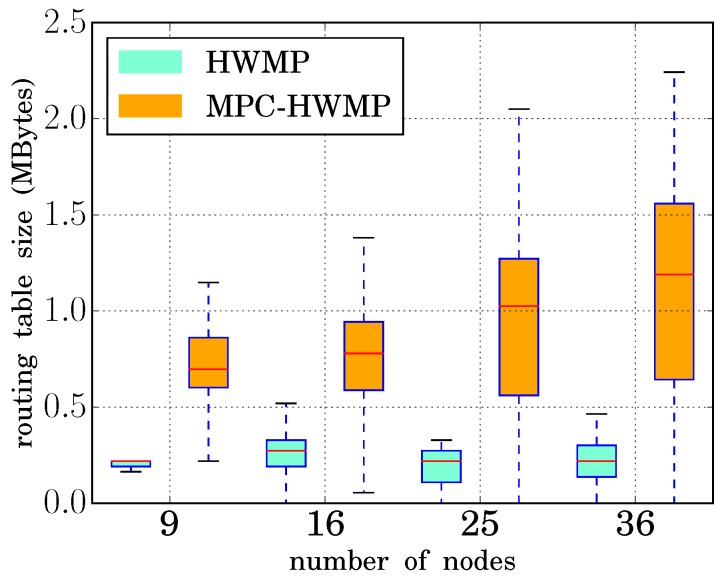
Routing table size (HWMP vs. MPC-HWMP).Smart grid architecture.

As explained above, to obtain the advantages offered by the multi-path mechanism, it was necessary to increase the number of entries that each node must store in the routing table, as well as to add some fields to those entries. Both actions translated into an increase in the amount of memory required in each node to store its routing table. To quantify this increase, the routing table size was also measured during the simulations. Figure 10 shows the results, both for the basic HWMP and for the modified protocol. For every network size, the minimum and maximum values are depicted (that is, the nodes with the smallest and largest table). Besides, the boxes represent the 25th, 50th, and 75th percentiles values.

When the basic HWMP was used, the routing table size was independent of the network size, since the nodes must only store the route to the data concentrator. However, with the multi-path modifications, the different available paths were stored to be assigned to the different traffic types. Thus, when the network size was increased, more paths became available, and so, the amount of memory needed for the routing table also grew. As shown in the figure, in the worst case taken into account in the simulations (36 nodes), the amount of memory needed (for the node with the largest table) was around 2.3 megabytes.

This fact could represent a scalability problem if the number of nodes in the network could grow indefinitely, but this is not the case with smart grid NANs, where one node represents one home. In any case, the amount of memory needed could reach the order of tens or hundreds of Mbytes, which with current memory technologies does not represent any problem.

#### 3.4.5. Control Channel Utilization Factor

The possibility of having multiple paths implies an increase in the number of PREQ and PREP packets that must be transmitted. To avoid the saturation of the data channels, we dedicated an exclusive channel to control packets. In order to evaluate the impact caused by these new transmissions, the measure of the control channel utilization factor (ρ) was also carried out. This parameter can be measured by each mesh station, the possible values being one (busy) or zero (idle). These values were then smoothed using an Exponentially-Weighted Moving Average (EWMA) in order to obtain an estimation of the average value and avoid abrupt oscillations.

**Figure 11:**
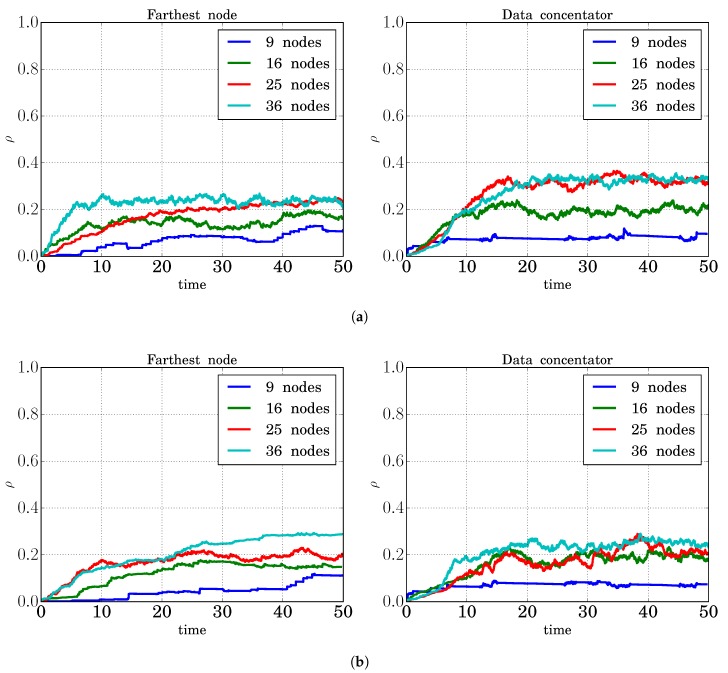
Control channel utilization factor. (**a**) Lifetime of reactive routing information: 2.05 s; (**b**) lifetime of reactive routing information: 5.12 s.

The results are shown in Figure 11, where the channel utilization factor for two cases (farthest node and the data concentrator), for two different values of the reactivePathTimeout parameter (lifetime of reactive routing information values) and for different network sizes (from 9–36 nodes) are presented. It can be observed how, as the network size was increased, more control packets were transmitted, and so, the ρ value also increased, for both reactivePathTimeout values. Besides, it is also shown that the ρ value did not achieve high values (it was always below 0.4), and therefore, the control channel was not congested.

## 4. Conclusions and Future Work

In this article, the implementation of smart grid NANs with multi-hop wireless mesh networks was considered. Specifically, a modification of the HWMP protocol was proposed and evaluated, based on the maintenance of multiple paths between each pair of network nodes. In addition, independent frequency channels were defined for each of the paths, as well as a special channel for control messages. With the joint application of both techniques, a more efficient utilization of the available network resources was achieved.

To evaluate the obtained benefits, the ns-3 network simulator was used, on which all the proposed modifications were implemented. The results of the simulations allowed us to verify the improvements in the network performance in terms of packet delivery ratio, throughput and network transit time. On the other hand, since the application of multipath techniques supposes an increase in the size of the routing tables stored in the nodes, the necessary amount of memory to store them was also measured. The results allow affirming that no memory problems will arise in the nodes.

As future lines of work, our research to improve NANs performance will continue by adding network security and data privacy. In this field, new extensions for the HWMP protocol based on multiparametric optimization techniques will be proposed, in order to take advantage of the improvements obtained with the multipath proposal and at the same time guaranteeing a better network service by prioritizing the paths through the nodes with a better reputation.

## Figures and Tables

**Table:** 

0.49	0.49
(a) t	(b) t

**Table 1:** Definition of the variables and functions for the PREQ and PREP mechanisms.

Parameter	Description
**preq** or **prep**	PREQ or PREP element received in the node.
**read**	Function that extracts the main fields of the **preq** or **prep** elements.
${\mathrm{OA}}_{C}$	Originator address of the current element received.
${\mathrm{OA}}_{P}$	Originator address of the previous element received.
${\mathrm{SN}}_{C_{\mathrm{OA}}}$	Sequence number of the current element received by ${\mathrm{OA}}_{C}$.
${\mathrm{SN}}_{P_{\mathrm{OA}}}$	Sequence number of the previous element received by ${\mathrm{OA}}_{P}$.
${\mathrm{DA}}_{C}$	Destination address of the element.
$m_{1\cdots n}$	Cumulative metrics of each path.
$f_{r}$	Retransmitter node.
PathID	Path identifier.
**updateMetric**	Calculate and update the airtime link metric according to the received **preq** or **prep** message.
**isValid**	Verify if the element received has updated information (sequence numbers), or if it has a better metric, or different $f_{r}$.
**updateTable**	Update or add a new route if the isValid function is true.
RouteSelection	Request a route to the destination (next hop), taking into account the application.
**createPrep**	Create the PREP message according to the parameters of the PREQ message.
**sendPrep**	Send the PREP unicast message to ${\mathrm{OA}}_{c}$.
${\mathrm{forward}}_{\mathrm{control}}$	Retransmit the **preq** broadcast message through all interfaces.

**Table 2:** New fields added to the HWMP routing table.

Field	Description
**Control channel metric**	Metric value of the control channel, obtained by the PREQ-PREP mechanism.
**Data channel metric**	Metric value of each data channel, obtained by the PREQ-PREP mechanism.
**Hop count**	Number of hops between two nodes.
**PathID**	Path identifier used by PREP messages.

**Table 3:** General simulation parameters.

Variable	Description	Value
**simulator**	Simulator	ns-3.28
**numNodes**	Number of nodes	from 9 to 36
**distanceNodes**	distance between nodes	80 m
**numRuns**	Number of runs	21
**simTime**	Simulation time	50 s
**transportLayer**	Transport layer	User Datagram Protocol (UDP)
**randomGenerator**	Random number generator	MRG32k3a

**Table 4:** Applications classification, distributions, and parameters. NL, Network Load.

Traffic Type	Applications	Packet Size		Packet Interval			EDCA
		Length (Bytes)	Distribution	Interarrival Time (s)		Distribution	
				NL1	NL2		
1	Demand Response, Outage Management.	60	Exponential	0.075	0.025	Exponential	Voice (Highest Priority: 1)
2	Video surveillance, Overhead Transmission Line Monitoring, Substation Automation Systems (SASs).	60	Exponential	0.075	0.025	Exponential	Video (Priority 2)
3	Home Energy Management (HEM), Electric Vehicles (EVs) Charging	512	Deterministic	0.075	0.025	Deterministic	Background (Priority 3)
4	Meter Data Management	512	Deterministic	0.075	0.025	Deterministic	Best Effort (Lowest Priority: 4)

**Table 5:** Mesh Peering Management (MPM) protocol parameters.

Variable	Description	Value
**maxRetries**	Maximum number of retries	4
**maxBeaconLoss**	Maximum number of lost beacons before the link will be closed	20
**maxNumberOfPeerLinks**	Maximum number of peer links.	4
**maxPacketFailure**	Maximum number of failed packets before the link will be closed	5

**Table 6:** Hybrid Wireless Mesh Protocol (HWMP) parameters.

Variable	Description	Value
**pathMode**	Path selection mode	On-demand
**maxQueueSize**	Maximum number of packets we can store when resolving the route	255
**maxPREQretries**	Maximum number of retries before we suppose the destination to be unreachable	5
**reactivePathTimeout**	Lifetime of reactive routing information	5.12 s

**Table 7:** Physical layer parameters.

Variable	Description	Value
**phyLayer**	Wireless physical layer.	802.11a
**controlChannelNumber**	Number of control channels.	1
**controlChannelFreq**	Frequency of the control channel.	5180 MHz
**dataChannelNumber**	Number of data channels.	4
**dataChannelFreq**	Frequency of data channels.	5200 MHz 5220 MHz 5240 MHz 5260 MHz
**propagationDelay**	Maximum propagation delay.	3.333
	Propagation loss model.	Log distance
**propagationModel**	Exponent: the exponent of the path loss propagation model.	3
	ReferenceDistance: the distance at which the reference loss is calculated (m).	1 m
	ReferenceLoss: the reference loss at the reference distance (dB) (the default is Friis at 1 m with 5.15 GHz).	46.667

## Abbreviations

The following abbreviations are used in this manuscript:

CBR	Constant Bit Rate
EDCA	Enhanced Distributed Channel Access
EV	Electric Vehicle
EWMA	Exponentially-Weighted Moving Average
HAN	Home Area Network
HWMP	Hybrid Wireless Mesh Protocol
MAC	Medium Access Control
MPC-HWMP	Multi-Path Multi-Channel Hybrid Wireless Mesh Protocol
MSTA	Mesh Station
MPM	Mesh Peering Management Protocol
NAN	Neighborhood Area Network
PDR	Packet Delivery Ratio
PREP	Path Reply
PREQ	Path Request
QoS	Quality of Service
SM	Smart Meter
UDP	User Datagram Protocol
WAN	Wide Area Network
